# Classical Hodgkin Lymphoma: A Joint Clinical and PET Model to Predict Poor Responders at Interim Assessment

**DOI:** 10.3390/diagnostics12102325

**Published:** 2022-09-26

**Authors:** Elizabeth Katherine Anna Triumbari, David Morland, Annarosa Cuccaro, Elena Maiolo, Stefan Hohaus, Salvatore Annunziata

**Affiliations:** 1 Nuclear Medicine Unit, TracerGLab, Department of Radiology, Radiotherapy and Hematology, Fondazione Policlinico Universitario A. Gemelli IRCCS, 00168 Rome, Italy; 2 Service de Médecine Nucléaire, Institut Godinot, 51100 Reims, France; 3 Laboratoire de Biophysique, UFR de Médecine, Université de Reims Champagne-Ardenne, 51100 Reims, France; 4 CReSTIC (Centre de Recherche en Sciences et Technologies de l’Information et de la Communication), EA 3804, Université de Reims Champagne-Ardenne, 51100 Reims, France; 5 Hematology Unit, Department of Radiology, Radiotherapy and Hematology, Fondazione Policlinico Universitario A. Gemelli IRCCS, 00168 Rome, Italy; 6 Hematology Unit, Center for Translational Medicine, Azienda USL Toscana NordOvest, 55100 Livorno, Italy; 7 Hematology Section, Department of Radiological Sciences and Hematology, Università Cattolica del Sacro Cuore, 00168 Rome, Italy

**Keywords:** classical Hodgkin lymphoma, ^18^F-FDG PET/CT, Deauville Score, early response assessment, total metabolic tumor volume, SUV_max_

## Abstract

(1) This study aimed to investigate whether baseline clinical and Positron Emission Tomography/Computed Tomography (bPET)-derived parameters could help predicting early response to the first two cycles of chemotherapy (Deauville Score at interim PET, DS at iPET) in patients with classical Hodgkin lymphoma (cHL) to identify poor responders (DS ≥ 4) who could benefit from first-line treatment intensification at an earlier time point. (2) cHL patients with a bPET and an iPET imaging study in our Centre’s records (2013–2019), no synchronous/metachronous tumors, no major surgical resection of disease prior to bPET, and treated with two cycles of ABVD chemotherapy before iPET were retrospectively included. Baseline International Prognostic Score for HL (IPS) parameters were collected. Each patient’s bPET total metabolic tumor volume (TMTV) and highest tumoral SUV_max_ were collected. ROC curves and Youden’s index were used to derive the optimal thresholds of TMTV and SUV_max_ with regard to the DS (≥4). Chi-square or Fisher’s exact test were used for the univariate analysis. A multivariate analysis was then performed using logistic regression. The type I error rate in the hypothesis testing was set to 5%. (3) A total of 146 patients were included. The optimal threshold to predict a DS ≥ 4 was >177 mL for TMTV and >14.7 for SUV_max_ (AUC of 0.65 and 0.58, respectively). The univariate analysis showed that only TMTV, SUV_max_, advanced disease stage, and age were significantly associated with a DS ≥ 4. A multivariate model was finally derived from TMTV, SUV_max_, and age, with an AUC of 0.77. (4) A multivariate model with bPET parameters and age at diagnosis was satisfactorily predictive of poor response at iPET after ABVD induction chemotherapy in cHL patients. More studies are needed to validate these results and further implement DS-predictive factors at baseline in order to prevent poor response and intensify therapeutic strategies a-priori when needed.

## 1. Introduction

Hodgkin lymphoma (HL) represents 0.4% of all new cancer cases in the United States every year, with an incidence of 2.6 per 100,000 people per year based on the 2015–2019 cases [1]. It is more frequently diagnosed among young people aged 20–34, while it more frequently causes death in patients older than 45 years old, with a peak of 24% mortality in the age range of 75–84 [1]. The 5-year relative survival rate is in continuous improvement and is currently settled at 89.1% [1]. Therefore, the present and future goals of the scientific community are concentrated on the identification of early biomarkers of disease aggressiveness to identify those remaining patients who will relapse or die despite current medical experience and treatment possibilities. However, a major difficulty that is often encountered by research groups is the limited sample size of the study cohorts.

Among clinical parameters, the International Prognostic Score for Hodgkin’s Lymphoma (IPS) is widely used, especially in patients with an advanced stage at diagnosis [2]; recently, programmed death ligand 1 (PD-L1) expression on peripheral-blood granulocytes has also been proposed as a prognostic factor in newly diagnosed HL [3], but larger studies are needed to avail its use in clinical practice.

The five-point Deauville Score (DS) was one of the first used predictive and prognostic factors with a prominent role in HL treatment setting and modification. It is already applicable after the first two induction cycles of chemotherapy [4,5] on interim-Positron Emission Tomography/Computed Tomography (iPET/CT) images for early response to treatment assessment. This score is based on the visual interpretation of residual tumor Fluorine18-Fluorodeoxyglucose (^18^F-FDG) uptake compared with two reference points: the mediastinum (i.e., blood pool) and the liver. During the standard course of primary treatment, patients presenting at response-to-treatment PET with residual ^18^F-FDG uptake higher than the liver at any of the initially involved sites, or with new FDG-avid sites, are considered poor or non-responders and are subjected to a treatment escalation [5,6].

Recent studies in the literature propose other clinical and PET/CT-derived markers for response to treatment [7,8,9], event-free survival [10,11], progression-free survival, and overall survival [12,13,14] prediction. ^18^F-FDG PET/CT textural and radiomic features were also demonstrated to be useful tools in lymphoma for histological prediction, prognostic assessment, and bone marrow involvement definition. However, the lack of methodological harmonization, defined reproducible cut-off values, and sufficiently large validation studies currently prevent the use of radiomics in clinical practice and integration in hematological guidelines [15,16].

Despite the fundamental role that PET/CT has demonstrated in the management of HL patients [5,10,17], no baseline PET parameter has been identified and introduced in the initial risk assessment algorithm to predict early response to treatment. However, a few studies showed a good predictive power of some baseline PET features with respect to iPET results [14,18,19,20].

Accordingly, the aim of this study was to investigate whether conventionally used baseline clinical and PET parameters could help, alone or in combination, to predict early response to chemotherapy in patients with classical Hodgkin lymphoma.

## 2. Materials and Methods

This study was approved by the Ethical Committee of Fondazione Policlinico Universitario A. Gemelli IRCCS (study code 3834) and all included subjects signed an informed consent form.

A retrospective data collection and analysis were performed for all consecutive patients who were diagnosed with HL at the Hematology Unit of our Institution between 2013 and 2019. The exclusion criteria were as follows: histological diagnosis of nodular lymphocyte-predominant lymphoma, absence of baseline ^18^F-FDG PET/CT (bPET) and interim PET/CT (iPET) images in our Centre’s records, presence of other synchronous/metachronous tumors, extensive surgical resection of HL disease for diagnostic purposes before bPET, first evaluation at disease relapse, and first two cycles of chemotherapy different from ABVD (doxorubicin hydrochloride (Adriamycin), Bleomycin sulfate, Vinblastine sulfate, and Dacarbazine).

### 2.1. Imaging Protocol

PET/CT studies were performed according to the European Association of Nuclear Medicine (EANM) guidelines [21]. Patients fasted for ≥6 h and their blood glucose levels were <200 mg/dL before the administration of 232 ± 42 MBq of ^18^F-FDG. Images were acquired at 60 ± 10 min of uptake time using a Gemini GXL (Philips Healthcare—LOR RAMLA reconstruction without PSF and TOF; 3 iterations, 33 subsets; 4 × 4 × 4 mm^3^ voxel size; Gaussian filter of 5 mm; reconstructed image matrix size of 128 × 128) or a Biograph mCT (Siemens Healthineers, Erlangen, Germany)—3D OSEM reconstruction with PSF modeling and TOF; 2 iterations, 21 subsets; 3.2 × 3.2 × 5 mm voxel size; application of Gaussian filter of 2 mm; reconstruction image matrix of 400 × 400) PET/CT scanner. A low-dose CT scan (120 kV, 50 mAs) was acquired similarly for both scanners, from the skull base to the mid-thighs, for anatomical localization of functional findings and attenuation correction. The reconstructed CT image had a matrix size of 512 × 512, a pixel size of 0.97 × 0.97 mm, and a slice thickness of 3 mm.

### 2.2. Data Collection

The collection of clinical data at baseline included all variables belonging to the IPS [22], classified as follows: sex (male or female), age (< or ≥45 years old), presence of B symptoms, stage (classified as limited for Ann Arbor stages I to IIB with no bulk, and advanced for Ann Arbor stages from IIB with bulk to IV), white blood cell count (< or ≥15.000/mm^3^), lymphocyte count (< or ≥600/mm^3^ and/or <8% of white blood cell count), hemoglobinemia (< or ≥10.5 g/dL), albuminemia (< or ≥4 g/dL), erythrocyte sedimentation rate (ESR) (classified in >30 mm/h associated with B symptoms, and >50 mm/h with no B symptoms), fibrinogen (< or ≥400 mg/dL), lactate dehydrogenase (< or > than normal range).

**Figure 1:**
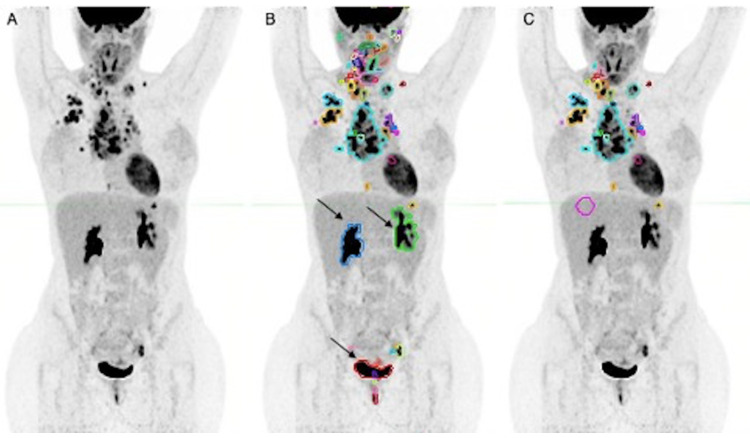
Total metabolic tumor volume (TMTV) calculation. Maximum intensity projection (MIP) views of a 32-year-old female patient with stage IVA nodular sclerosing Hodgkin lymphoma. (**A**) Unprocessed MIP. (**B**) Semiautomatic segmentation of the whole-body using LesionID^®^ tool from MIM Encore Software (version 7.0.5, MIM Encore Software Inc., Cleveland, OH, USA); some contoured physiological sites of uptake (e.g., black arrows) were later excluded from the analysis. (**C**) Revised contouring by two nuclear medicine physicians in consensus, which was then processed by the software to obtain the TMTV volumetric parameter (105.05 mL). The pink circle in (**C**) is the region of interest on the right liver lobe for PERCIST-based cut-off criterion for volumes of interest determination (see text for more detail).

PET data were collected by two nuclear medicine physicians in consensus. In detail, the LesionID^®^ tool from MIM Encore Software (version 7.0.5, MIM Encore Software Inc., Cleveland, OH, USA) was used to calculate each patient’s total metabolic tumor volume (TMTV) at bPET [23]. The preset PET Response Criteria in Solid Tumors (PERCIST)-based cut-off criterion for volumes of interest (VOI) determination was 41% of the maximum standardized uptake value (SUV_max_) of a 2 cm region of interest drawn on the right liver lobe; a semiautomatic VOI was then automatically drawn by the software on all sites (nodal and extra-nodal) of matching uptake characteristics in the whole-body 3D image of each patient. Physicians undertook a careful review to determine whether each contoured site of uptake was malignant or benign to exclude the latter from the analysis. All contoured segments were interpreted by evaluating ^18^F-FDG uptake on the PET and fused images, and anatomy on the CT images. Liver, lung, bone marrow, and spleen were considered involved in case of focal uptake. The TMTV volumetric parameter was obtained through software processing and recorded for each patient. An example of how the whole TMTV contouring process was performed is provided in Figure 1.

The highest maximum SUV_max_ detected from tumor sites on bPET and the DS from iPET evaluation were also recorded for each patient. Early response to chemotherapy was considered as a complete response if the DS at iPET after induction chemotherapy was <4. Poor response (comprising stable disease, progression of disease, and partial response) was defined as iPET DS ≥ 4.

### 2.3. Statistical Analysis

For the descriptive analysis, qualitative variables were described using absolute numbers and percentages. Quantitative variables were described using mean, standard deviation, median, quartiles, and extreme values.

Statistical analyses were performed using XLSTAT software (Addinsoft, Paris, France). Receiver operating characteristic (ROC) curves and Youden’s index were used to derive optimal thresholds of TMTV and SUV_max_ with regard to the Deauville Score (1–2–3 vs. 4–5). The area under the ROC curve (AUC), sensitivity, specificity, and corresponding 95% confidence interval (95% CI) were reported. Univariate analyses were conducted using chi-square or Fisher’s exact tests when appropriate. A multivariate analysis using logistic regression (binary logit) was performed using a variable selection procedure based on the *p*-value. The type I error rate in hypothesis testing was set to 5%.

## 3. Results

**Figure 2:**
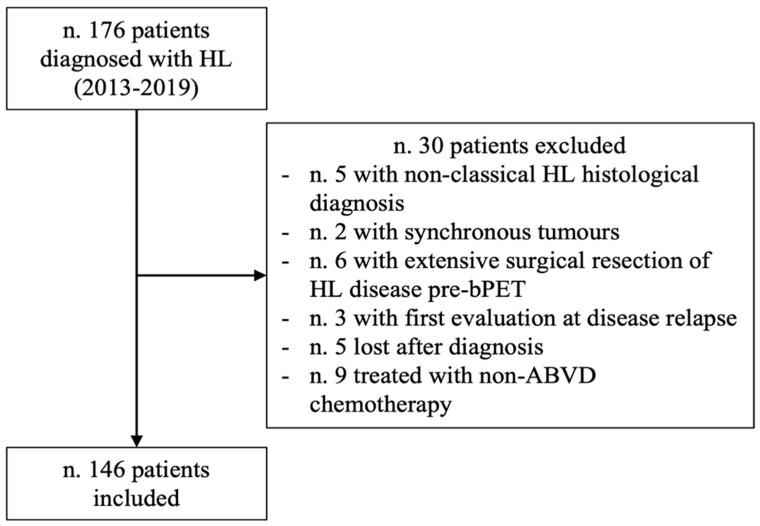
Flowchart of the study population.

Among 176 patients consecutively diagnosed with HL, 146 met the inclusion criteria and were considered for further analysis (Figure 2).

Table 1 and Table 2 display the patients’ clinical and PET variables, respectively. The performance of bPET exams was found to be fairly balanced between the Gemini GXL scanner (72/146, 49%) and the Biograph mCT (74/146, 51%).

The optimal threshold for TMTV and SUV_max_ to predict poor responders at iPET based on the DS were >177.02 mL and >14.67, respectively, with a sensitivity of 70% (95% CI, 61.2 to 77.5) for TMTV and 53.3% (95% CI, 44.4 to 62.0) for SUV_max_, and a specificity of 61.5% (95% CI, 42.5 to 77.5) for TMTV and 80.8% (95% CI, 61.5 to 91.8) for SUV_max_. The AUC reached 0.65 for TMTV and 0.58 for SUV_max_.

At univariate analysis, only TMTV > 177.02 mL, SUV_max_ > 14.67, advanced disease stage and age ≥45 years old were significantly associated with a DS ≥ 4 at iPET, while all other baseline clinical variables did not have significant predictive power (Table 3).

**Figure 3:**
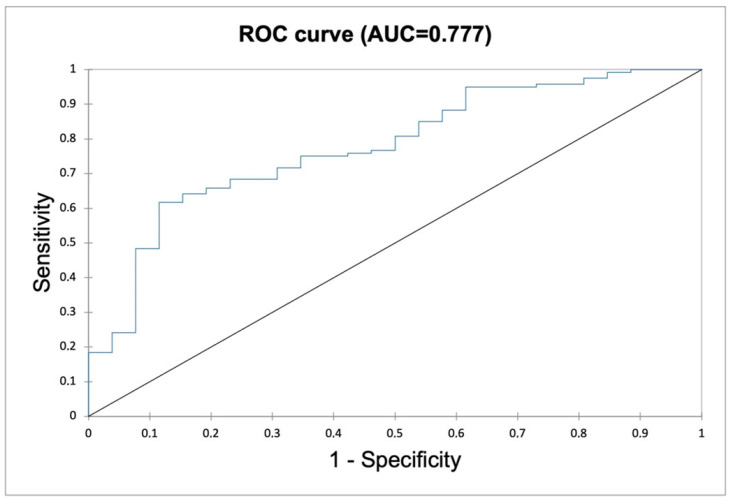
Multivariate model (TMTV + SUV_max_ + age) accuracy.

At multivariate analysis, the model derived from the three significant parameters TMTV, SUV_max_, and age had an AUC of 0.77 (95% CI, 0.68–0.86) (Figure 3). The “advanced stage” parameter only reached a *p*-value of 0.067. However, the possibility of its inclusion in a multivariate model together with TMTV, SUV_max_, and age was tested, and the AUC did not significantly improve, reaching 0.79 (95% CI, 0.71–0.88).

## 4. Discussion

The ability to anticipate HL patients’ risk assessment and stratification from iPET to bPET would be crucial given that 20–30% of patients still relapse or die despite the advancements in therapeutic strategies and optimal use of the DS [6,22].

In this study, we found that a multivariate model stratifying patients by age > 45 years old, TMTV > 177.02 mL, and highest tumoral SUV_max_ > 14.67 could identify to a certain extent those who will have a poorer response to chemotherapy at early response assessment (iPET DS ≥ 4). The univariate analysis showed a significant association between each of these three parameters and DS ≥ 4 at iPET, but with rather low sensitivity and specificity and an AUC not higher than 0.65. A combination of the three parameters demonstrated a higher predictive performance. If further studies will confirm such results, the introduction of such a model in the baseline risk assessment of cHL patients could improve the rate of patients with complete response at iPET, as it would suggest intensifying first-line chemotherapy at an earlier time point and could lead to a favorable DS at iPET.

Few other studies in the literature investigated the value of baseline PET parameters to predict early response to primary chemotherapy in patients with HL [18,19,20,24]. In a study on a pediatric HL population, Rogasch et al. found that among other PET-derived metabolic and heterogeneity parameters, TMTV had the best predictive power for inadequate response to induction chemotherapy [19]. Ben Bouallègue and colleagues investigated whether bPET metabolic, textural, and morphological tumoral indices were predictive of early metabolic response at iPET in a cohort of 57 patients with HL (25% of the population) and non-HL (NHL, 75%) bulky malignant lymphomas and found positive results, suggesting that these parameters could be valuable tools for further assessment of tumor aggressiveness and forecasting sensitivity to chemotherapy [18]. In contrast, Pike et al. conducted a study on a population of patients with advanced HL and found that baseline total lesion glycolysis and metabolic tumor volume (MTV) of the bulkiest lesion were significantly associated with iPET response [20]. The MTV of the bulky lesion was also the most relevant feature and deemed to add significant prognostic insight to the interim PET response assessment in a study by Kanoun et al. [24].

These studies had a similar aim to the study we present here, although they considered a pediatric population or a heterogeneous HL and NHL population, as well as the largest tumoral site. Our work was focused on cHL adult patients and took into account a smaller range of PET parameters, but the cohort was homogeneous with respect to the undertaken chemotherapy and a considerable number of other baseline clinical variables were considered. TMTV and SUV_max_ were chosen to have an estimate of the patient’s tumor burden and an easily reproducible tumoral metabolic index, respectively. On the one hand, the predictive capabilities of TMTV described in the literature are confirmed here; on the other, the added value of SUV_max_ needs further studies in cHL settings to be confirmed.

Unexpectedly, among the clinical parameters considered for our analysis, only age had a significant association with the outcome. Clinical variables were chosen as belonging to the well-known IPS. This score is availed in clinical practice for patients with advanced HL. We adjusted the IPS stage parameter by considering all Ann Arbor stages and not strictly stages III/IV. However, it was associated with the DS at iPET at univariate analysis only (*p*-value 0.02 at univariate analysis vs. 0.067 at multivariate analysis; Table 3). It was, however, tested in a multivariate model together with the significant parameters TMTV, SUV_max_, and age, leading to a non-significant improvement of the model’s accuracy (AUC of 0.79 vs. 0.77). Overall, other published results suggesting the decrease in the meaning of the IPS at the time of PET-guided treatments [25] were confirmed here.

Some considerations can be made regarding the chosen outcome. It is well known that the DS is the best instrument to evaluate response to treatment, and despite new tools being suggested, it still holds its primacy [26]. To be able to predict the DS would mean to anticipate therapeutic modifications as soon as possible. On the other hand, iPET DS is an image-derived marker of disease status compared with baseline disease burden, while other outcomes, such as overall, event-free, and progression-free survival depend on objective data and are not influenced by the data collection methodology or PET/CT scanner type [16]. However, in retrospective studies, a significant number of events would be needed for data analysis, and the overall good prognosis of cHL patients hinders the goal. Our population had not yet undergone a long-term follow-up, with a consequent very low number of events counted and the impossibility of performing a survival assessment.

As the prognostic role of other clinical, radiomic, textural, and genomic features at baseline will be confirmed in cHL patients, it is very well hoped that new and more complete models will arise given that the analysis of big datasets and hidden meanings among their parameters is evermore entrusted to artificial intelligence.

Some limitations to this study subsist. First, its retrospective, single-center nature, which may have introduced inclusion biases, despite all exclusion criteria being applied to avoid population heterogeneity. Second, even if quite a numerous cohort was recruited compared with other cHL studies in the literature, we recognize it is still limited for our results to be generalizable. Therefore, further larger prospective studies are needed. Finally, the absence of a validation cohort is of note, though we were not able to gather it in a reasonable number so to be considered, and could be the object of a future study.

## 5. Conclusions

The ability to predict a cHL patient’s response to induction chemotherapy could allow for choosing treatment intensification a priori. This retrospective study found a multivariate model that combined patients’ TMTV, SUV_max_ and age with good predictive power. Further larger prospective studies and validation of the model are needed for the generalizability of the obtained results.

## Figures and Tables

**Table 1:** Baseline clinical characteristics of the cohort (n = 146).

Patients’ Characteristics	n (Range)	%
Sex		
Female	82	56
Male	64	44
Age		
Mean (range)	39 (16–82)	
<45 years old	106	73
≥45 years old	40	27
B symptoms		
No	86	59
Yes	60	41
Disease stage		
Limited (I—IIB with no bulk)	70	48
Advanced (IIB with bulk—IV)	76	52
White blood cell count		
<15.000/mm^3^	124	85
≥15.000/mm^3^	22	15
Lymphocyte count		
<600/mm^3^ and/or <8% of white blood cell count	3	2
≥600/mm^3^	143	98
Hemoglobinemia		
<10.5 g/dL	29	20
≥10.5 g/dL	117	80
Albuminemia		
≥4 g/dL	73	50
<4 g/dL	73	50
Erythrocyte sedimentation rate		
>30 mm/h, with B symptoms	86	59
>50 mm/h, without B symptoms	60	41
Fibrinogen		
<400 mg/dL	42	29
≥400 mg/dL	104	71
Lactate dehydrogenase		
<Normal range	122	84
>Normal range	24	16

**Table 2:** Baseline and interim PET parameters considered for the whole cohort (n. 146).

PET Parameter	Values
**TMTV (*bPET*)**	
Mean value ± DS	218.80 ± 249.37
Median (1st–3rd Quartile)	116.91 (47.52–312.10)
Range	0.73–1145.68
**SUV_max_ (*bPET*)**	
Mean value ± DS	16.40 ± 8.31
Median (1st–3rd quartile)	15.33 (10.88–20.16)
Range	2.55–66.0
**Deauville Score (*iPET*)**	
<4	
No. of patients (%)	120 (82)
≥4	
No. of patients (%)	26 (18)

TMTV: total metabolic tumor volume; bPET: baseline positron emission tomography; SUV_max_: maximum standardized uptake value; iPET: interim positron emission tomography (after induction chemotherapy).

**Table 3:** Univariate and multivariate analysis.

Variable	Univariate *p*-Value	Multivariate *p*-Value
**TMTV** > 177.02 mL	0.002 *	0.013 *
**SUV_max_** > 14.67	0.002 *	0.002 *
**Stage** (advanced)	0.022 *	0.067
**Age** ≥ 45 years	0.045 *	0.05 *
**Sex**	0.478	Excluded
**WBC** ≥ 15,000/mm^3^	0.222	Excluded
**Symptoms** (present)	0.942	Excluded
**Albuminemia** ≥ 4 g/dL	0.287	Excluded
**Fibrinogen** ≥ 400 mg/dL	0.718	Excluded
**Erythrocyte sedimentation rate** (>30 mm/h with B symptoms vs. >50 mm/h without)	0.714	Excluded
**Hemoglobinemia** < 10.5 g/dL	0.899	Excluded
**Lymphocyte count** < 600/mm^3^ or <8% of WBC	0.896	Excluded
**LDH** > normal range	0.846	Excluded

TMTV: total metabolic tumor volume; SUV_max_: maximum standardized uptake value; Stage (advanced): Ann Arbor stages from IIB with bulk to IV; WBC: white blood cell count; LDH: lactate dehydrogenase; *: *p* ≤ 0.05.

## Data Availability

The data presented in this study are available on request from the corresponding author. The data are not publicly available due to privacy regulations.
